# Emergency contraceptive pill awareness in Bangladesh: missed opportunities in antenatal care and family welfare assistant visits

**DOI:** 10.1186/s12978-024-01922-x

**Published:** 2024-12-18

**Authors:** Tasnim Ara, Shahnaj Sultana Sathi, Shafayatul Islam Shiblee, Sumaiya Nusrat Esha, Md Tazvir Amin, Md Mahabubur Rahman

**Affiliations:** 1 Institute of Statistical Research and Training, University of Dhaka, Dhaka, Bangladesh; 2 International Center for Diarrhoeal Disease and Research, Bangladesh, icddr,b, GPO Box 128, Dhaka, 1000 Bangladesh

**Keywords:** Emergency contraceptive pills, Family planning, Awareness, Antenatal care, Family welfare assistance, Provider bias, Bangladesh

## Abstract

**Background:**

Despite high coverage of antenatal care (ANC) and family welfare assistant (FWA) visits, emergency contraceptive pill (ECP) awareness is critically low in Bangladesh. We aim to investigate the missed opportunities in generating ECP awareness through ANC and FWA visit programs; and assess the missed opportunities and sociodemographic discrimination in receiving family planning (FP) counseling during ANC.

**Methods:**

We used data from the nationwide Bangladesh Demographic and Health Survey 2017–18. Sample includes 5012 reproductive-aged women who gave live birth in the last 3 years preceding the survey. We used mixed-effect multiple logistic regression considering women nested within clusters to conclude.

**Results:**

Nationally, 79% of women who gave live birth in the last 3 years preceding the survey were unaware of ECP. The estimated missed opportunities in generating ECP awareness was 59.5% in ANC, 0.9% in FWA visits, and 12.3% in both ANC and FWA visits. While FWA visit was not associated with ECP awareness, receiving FP counseling during ANC was significantly associated. About 88.4% of women remained unexposed to FP counseling through ANC during their last pregnancy. Missed opportunities in FP counseling during ANC was 80.4% of which 72% points were from qualified providers. The odds of missed opportunities was not associated with provider type, rather significantly increased among women with low education, lower parity, and poor socioeconomic status.

**Conclusions:**

This study highlights the fragile status of FP counseling during ANC and FWA visits in generating ECP awareness. A prominent provider bias is excluding women of the disadvantageous sociodemographic group from receiving FP counseling.

## Abstract (plain language summary)

Emergency contraceptive pill (ECP) awareness is low in Bangladesh, particularly among women with low education, and poor socioeconomic status. However, the extent to which the antenatal care (ANC) and family welfare assistant (FWA) programs fail to generate ECP awareness is never measured.

To address this knowledge gap, we estimated the missed opportunity to generate ECP awareness through ANC and FWA programs. Further, we investigated the missed opportunity and sociodemographic discrimination in receiving family planning counseling during ANC. We scrutinized the Bangladesh Demographic and Health Survey 2017–18 data of 5012 women who gave their last live birth 3 years preceding the survey.

About 73% of women remain unaware of ECP despite receiving ANC or getting FWA visits at home. Family planning counseling during ANC increased ECP awareness but the FWA program did not. Women from rural areas, with low education, limited media exposure, and from lower wealth quintiles are still left unaware of ECP. The missed opportunity of providing family planning counseling during ANC is high (80.4%), particularly among women with low education, lower parity, and poor socioeconomic status.

The study provides valuable insights to the policy planners for rectifying the family planning program of ANC and FWA visits in Bangladesh. Findings reveal the disadvantaged women who know less about ECP and are informed less about family planning during ANC which may help programs to reach the unreached women.

## Background

Unintended conception has detrimental consequences on mothers and newborns. Women with unintended conception are less likely to seek essential maternity care, and more likely to have pre-term birth, and adverse psychological outcomes [1–6]. Newborns of unintended conception have a greater risk of being born with low birth weight and dying during infancy [1–6]. Beyond maternal and neonatal outcomes, unintended conception’s consequences affect the entire society’s well-being through adverse social, health, and economic outcomes [6–8].

Bangladesh, the third largest populous country in South Asia and eighth in the world is suffering from 0.27 million (estimated) unwanted births and 0.43 million (estimated) mistimed births every year [9, 10]. Many mistimed and unwanted conceptions often end with abortion which accumulates to a total of 1.2 million abortions every year in Bangladesh [11]. As a result, Bangladesh is now struggling to reduce its TFR which has stagnant at the level of 2.3 since 2011 [12, 13]. In Bangladesh, mistimed births primarily occur among women of young age (< 24 years) and first or second parity, and unwanted births mostly occur at late reproductive age (> 30 years) with high parity [10]. Fifty percent of women in Bangladesh completed their child desire by the age of 26 years and fifty percent underwent menopause by the age of 46 years [10]. This suggests on average, a Bangladeshi woman remains at risk of unwanted birth for 20 years. Uninterrupted FP use throughout these years becomes troublesome because ensuring continuous availability and compliance are challenging.

To avert an unintended conception a woman needs to choose an appropriate FP method based on her child-desire. However, Bangladeshi women are not adopting methods based on their child-desire. For example: women who do not want more children mainly use short-acting modern methods (48%) and traditional methods (13%), or no method (15%). As short-acting methods are widely used, emergency contraceptive pill (ECP) can be a supportive measure alongside the short-acting family planning (FP) methods aimed at averting unwanted births [14]. ECP can be used if there is unprotected intercourse or concerns about possible contraceptive failure from non-compliance such as condom breakage, slippage, 3 or more consecutively missed combined oral contraceptive pills, or 3 days late during the first week of the cycle, more than 7 days late for the combined injectable contraceptive, miscalculation of the abstinence period, failed withdrawal. In such cases, if ECP is administered within 72 h, it exhibits an 89% efficacy rate in averting unintended conception [15].

The World Health Organization (WHO) recommended that all women and girls have a right to access emergency contraception and these methods should be routinely included within all national family planning programs [16]. In line with the WHO recommendation, Bangladesh’s national family planning programs have the provision of counseling on ECP. However, ECP awareness remains critically low (18%) [10], which demands scrutinizing the FP programs. FP counseling has already been introduced as one of the key components of antenatal care (ANC). Hence, the high coverage of ANC (92% received at least one ANC [10]) provides an excellent opportunity to counsel women about ECP. In addition, Bangladesh’s FP program provides door-to-door FP counseling by family welfare assistants (FWAs) [17]. FWA program was first introduced in 1976 [18]. FWAs are permanent female staff under the Directorate General of Family Planning (DGFP) who must have at least 10 years of formal schooling. They are supposed to visit each household in their catchment area once every 2 months. During their visits, they are obliged to register new couples and update the couple registers containing information about all currently married women ages 15–49 and their husbands in their catchment area. The primary responsibilities of FWAs include counseling couples on FP, distributing oral contraceptive pills, condoms and misoprostol, and referring couples to appropriate health facilities if they express interest in long-acting reversible contraceptives or permanent methods.

Despite the high coverage of ANC and FWA visits, ECP awareness is low with large sociodemographic heterogeneity [10] demanding investigation of missed opportunities for generating ECP awareness in ANC and FWA visits. To authors best knowledge, only two national-level studies had identified the correlates of ECP awareness in Bangladesh [19, 20]. None of these studies focused on the missed opportunities in ECP knowledge generation during ANC and FWA visits which are essential in bridging the sociodemographic inequity in ECP awareness. Therefore, this study aims to investigate the missed opportunities in generating ECP awareness through ANC and FWA visit programs. Furthermore, we aim to assess the missed opportunity and sociodemographic discrimination in receiving FP counseling during ANC.

## Materials and methods

### Data, setting, and participants

**Fig. 1:**
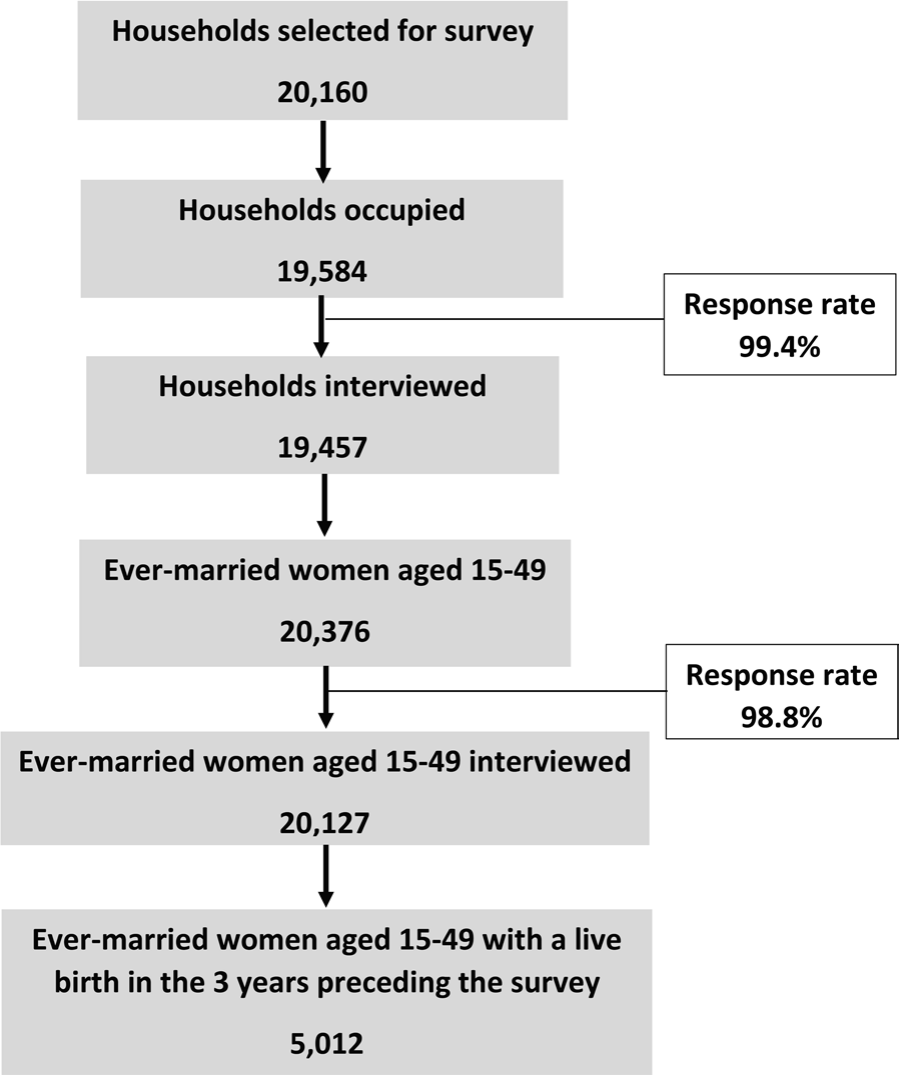
Data flow of the analytical sample

We used secondary data from the nationally representative cross-sectional Bangladesh Demographic and Health Survey (BDHS) 2017–2018. The BDHS 2017-18 data was collected using two-stage stratified cluster sampling making the data representative at the administrative division level, as well. As we aimed to examine the missed opportunities in ECP awareness and family planning counseling by FWA and ANC visits, we conducted the analyses on all women (5012) aged 15–49 who gave their most recent live birth 3 years preceding the survey. Figure 1 presents the data flow of the analytical sample.

### Outcome measures

***Outcome variable 1:*** ECP awareness was the first outcome variable with two categories: aware of ECP (if a woman ever heard about ECP from any source) and unaware of ECP (if a woman never heard about ECP from any source).

***Outcome variable 2:*** We constructed the second binary outcome variable- “missed opportunity in providing FP counseling during ANC” as follows:

- missed opportunity (if women had ANC visits but did not receive FP counseling during ANC),
- utilized opportunity (if women had ANC visits and received FP counseling during ANC).

### Conceptual framework and covariates

**Fig. 2:**
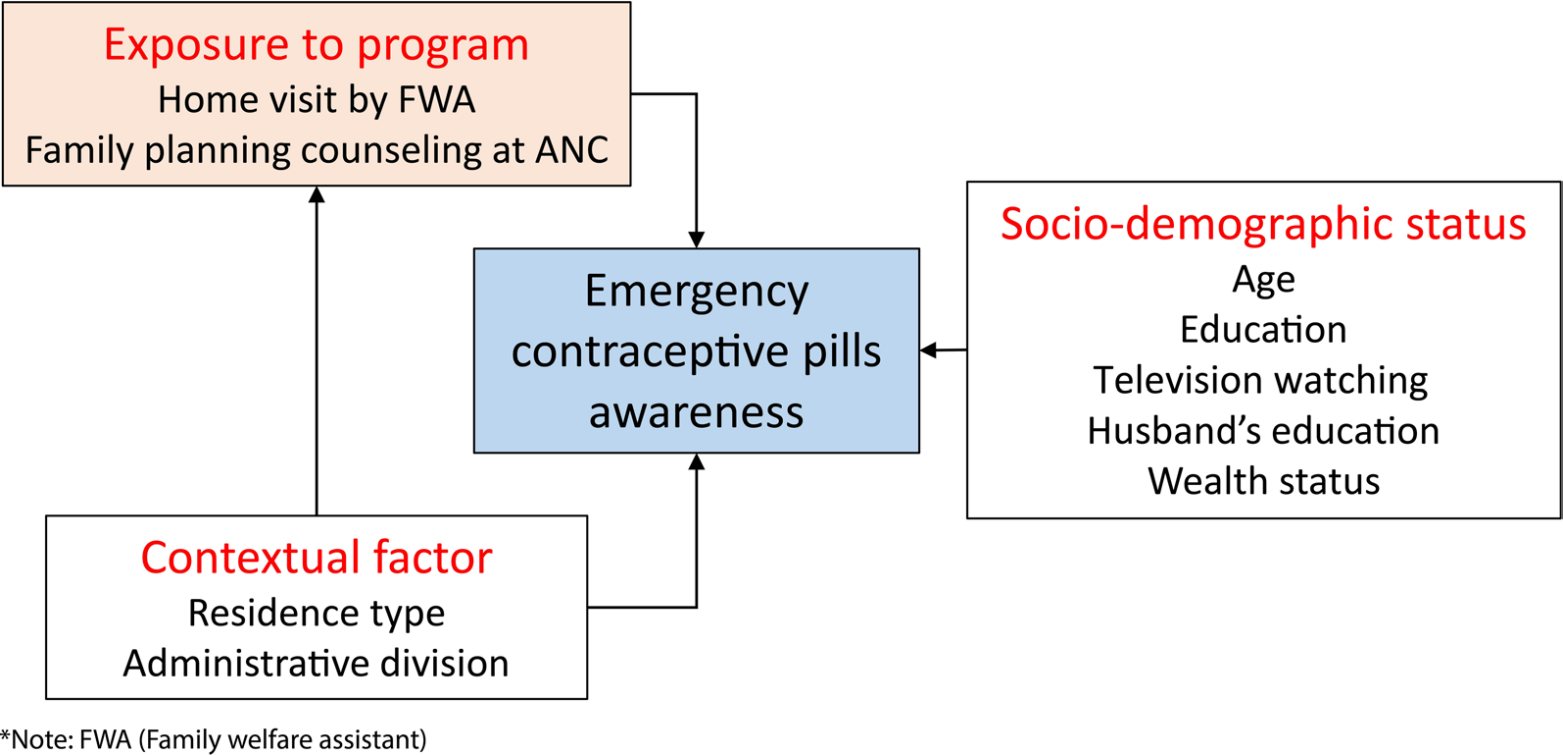
Conceptual framework of emergency contraceptive pills awareness

***Conceptual framework for outcome 1:*** We conceptualize that ECP awareness can be influenced by three broad domains: exposure to the FP program, contextual factors, and socio-demographic characteristics (Fig. 2). If a woman is exposed to FP programs like FWA or ANC visits, she may have a higher chance of knowing about ECP than a woman who is unexposed to such programs. Contextual factors shape customs and beliefs of the community which may further influence women’s knowledge about FP methods. For example: A community with conservative customs and beliefs will have less discussion about FP than a community where people are concerned about FP rights and choices. Thus, conservative customs of the community may shape women’s perception and urge of FP which may lessen the chance of knowing about ECP. Contextual factors may further influence ECP awareness through varying intensities of FP programs because FP programs of a region are designed based on the needs of that region. Women from advantageous socio-demographic groups (i.e. better education and wealth, frequent media exposure) may have higher ECP knowledge because of their better exposure to health services and their desire to limit family size.

***Covariates for modeling outcome 1:*** Under each domain, we selected covariates based on earlier studies from Bangladesh [19, 20], Nepal [21], India [22, 23] and Sub-Saharan Africa [24]. The details of the covariates are presented in Table 1. The wealth status was constructed using a principal component analysis based on household goods, which ranged from having a television to having a bicycle or a car, and households’ sources of drinking water, sanitation facilities, and flooring materials. Detailed about household wealth can be found in BDHS 2017–18 final report.

**Table 1:** Covariate construction: Definition of all the categories under each covariate

Covariates	Categories	Definition
***Exposure to the FP program***		
Got FWA Visit	No	Woman did not get FWA visit at home in 6 months preceding the survey
	Yes	Woman got FWA visit at home in 6 months preceding the survey
FP counseling during ANC	Had no ANC visits during last birth	Woman did not receive ANC during last birth
	Didn’t receive FP counseling during ANC	Woman did not receive FP counseling during ANC for last birth
	Received FP counseling during ANC	Woman received FP counseling during ANC for last birth
***Contextual factors***		
Type of residence	Urban	Reside in urban areas
	Rural	Reside in rural areas
Administrative division	Dhaka	Reside in Dhaka division
	Chattogram	Reside in Chattogram division
	Barishal	Reside in Barishal division
	Khulna	Reside in Khulna division
	Mymensingh	Reside in Mymensingh division
	Rajshahi	Reside in Rajshahi division
	Rangpur	Reside in Rangpur division
	Sylhet	Reside in Sylhet division
***Socio-demographic characteristics***		
Women’s current age	15–19	Woman aged 15–19 years
	20–24	Woman aged 15–19 years
	25–29	Woman aged 25–29 years
	30–34	Woman aged 30–34 years
	35–49	Woman aged 35–49 years
Women’s education	No education or primary	No formal education or completed primary-level education only
	Secondary incomplete	Ever attended class 6–10 but did not complete class 10
	Secondary complete or higher	Completed at least class 10
Watching Television	Not at all	Does not watch television
	Less than once a week	Watches television less than once a week
	At least once a week	Watches television at least once a week
Husband’s education	No education or primary	No formal education or completed primary-level education only
	Secondary incomplete	Ever attended class 6–10 but did not complete class 10
	Secondary complete or higher	Completed at least class 10
	Unknown	Husband's educational information is unknown
Household wealth quintile	First	Household belonged to the first wealth quintile group
	Second	Household belonged to the second wealth quintile group
	Third	Household belonged to the third wealth quintile group
	Fourth	Household belonged to the fourth wealth quintile group
	Fifth	Household belonged to the fifth wealth quintile group
***Additional covariates for modeling outcome 2***		
Type of ANC provider	Medically trained provider	Qualified doctor, nurse/midwife/paramedic, family welfare visitor, community skilled birth assistant, and sub-assistant community medical officer
	Non-medically trained provider	Otherwise
Parity	First	Ever born one child
	Second	Ever born two children
	Third or higher	Ever born three or more children

***Conceptual framework for outcome 2:*** We conceptualized that missed opportunities for FP counseling during ANC can be shaped by two broad domains: providers’ characteristics and women’s characteristics. Providers’ perceptions about FP counseling during ANC can be one of the key drivers of counseling. In regards, medically trained providers (MTP) are likely to hold a more positive attitude towards post-partum FP counseling during ANC than non-MTP, maybe because of receiving more training on ANC services. As we aimed to explore whether the providers are being selective in providing FP counseling during ANC, we included broad sociodemographic groups as covariates. As TFR is comparatively high among women from disadvantageous socioeconomic groups like low education, rural residents, and lower wealth quintiles, they may have a higher chance of not receiving FP counseling during ANC. We also hypothesized that women with first or second parity may be less likely to be counseled because providers may emphasize higher parity cases for FP counseling [25, 26].

***Covariates for modeling outcome 2:*** Under the domain provider’s characteristics, BDHS 2017–18 data only allows to include provider type (MTP or non-MTP). BDHS 2017–18 considered qualified doctors, nurses/midwives/paramedics, family welfare visitors, community skilled birth assistants, and sub-assistant community medical officers as medically trained providers. Under sociodemographic characteristics, we included women’s education, number of children ever born, type of residence, administrative division, and wealth quintile.

### Statistical analysis

#### Analysis under objective 1

Firstly, we estimate the missed opportunities in ANC and FWA visits for generating ECP awareness at the national level and then at the divisional level to explore regional variation. Estimation procedures were as follows:

***Missed opportunity in ANC visits in generating ECP awareness:*** This was measured as the percentage of women who had ANC visits in their most recent live birth but never heard about ECP among women who gave their last live birth in 3 years preceding the survey.

***Missed opportunity in FWA visits in generating ECP awareness:*** This was measured as the percentage of women who had FWA visits in the last 6 months preceding the survey but never heard about ECP among women who gave their last live birth in 3 years preceding the survey.

For measuring missed opportunity we used “all women who gave live birth in 3 years preceding the survey” as the denominator because this will reflect where FP awareness could reach if all contact points could reach their highest mark.

Secondly, we estimated the prevalence of ECP awareness across the factors considered under each domain. As BDHS 2017–18 used a cluster sampling design, we used a mixed-effect multiple logistic regression model considering random intercept at the cluster level to examine the factors associated with ECP awareness. The random intercept model can deal with the hierarchical nature of the clustered data by incorporating both fixed effects and random effects at the cluster level. These random effects capture the variation across clusters by explicitly modeling the clustering structure and fixed effects represent the average relationship between the predictors and the response variable across all clusters.

#### Analysis under objective 2

Firstly, we estimated the missed opportunities for FP counseling during ANC by providers’ type (medically trained and untrained) at the national level and then at the divisional level to explore regional variation. Missed opportunity in FP counseling during ANC was estimated as the percentage of women who had ANC visits in their most recent live birth but did not receive FP counseling among women who gave their last live birth 3 years preceding the survey. For measuring missed opportunity we used “all women who gave live birth in 3 years preceding the survey” as the denominator because this will reflect where FP awareness could reach if all contact points could reach their highest mark.

Secondly, we measured missed opportunities among “women who had ANC visits for their most recent birth” to understand whether the providers are being selective in providing FP counseling during ANC. We also used a mixed-effect multiple logistic regression considering women nested within clusters to examine the factors associated with missed opportunities in FP counseling during ANC.

To reduce bias, we incorporated appropriate sampling weights that adjusted for the complex survey design characteristics of BDHS. For all the statistical analyses, we used Stata V.14.0 (Stata SE V.14, Stata Corp., College Station, Texas, USA).

## Results

### Sample characteristics

About 53% of women were aged 15–24, only 6% were uneducated, and more than one-third did not watch television, 13.6% women’s husbands were uneducated, and 73% were from rural areas (Table 2). Only 16.4% got FWA visits, 82% received ANC from MTP, and 11.5% received FP counseling during ANC.

**Table 2:** Percentage distribution of women across different sociodemographic groups and program exposure

Factors	Number of women	Percentage distribution of women
Overall	5012	100.0
Women’s current age		
15–19	869	17.9
20–24	1773	35.2
25–29	1310	25.9
30–34	749	15.1
35–49	311	5.9
Women’s education		
No education	312	6.3
Up to primary complete	1392	27.6
Secondary incomplete	2136	43.7
Secondary complete or higher	1172	22.4
Watching television		
Not at all	1907	36.4
Less than once a week	442	9.3
At least once a week	2663	54.3
Husband’s education		
No education	679	13.6
Up to primary complete	1657	33.6
Secondary incomplete	1366	28.0
Secondary complete or higher	1231	23.8
Unknown	79	1.5
Parity		
First	1915	38.2
Second	1638	32.8
Third or higher	1459	29.0
Household wealth quintile		
First	1079	20.6
Second	1017	20.5
Third	905	19.2
Fourth	988	20.2
Fifth	1023	19.5
Type of residence		
Urban	1725	26.8
Rural	3287	73.2
Administrative division		
Dhaka	741	25.6
Barishal	533	5.7
Chattogram	835	21.2
Khulna	524	9.2
Mymensingh	603	8.5
Rajshahi	527	11.6
Rangpur	559	10.6
Sylhet	690	7.6
Got FWA visit		
No	4233	83.6
Yes	779	16.4
Type of ANC provider		
Medically trained provider	4100	81.9
Non-medically trained provider	504	10.1
Had no ANC during last birth	408	8.0
FP counseling during ANC		
Didn’t receive FP counseling during ANC	4011	80.5
Received FP counseling during ANC	593	11.5
Had no ANC during last birth	408	8.0

### Missed opportunities to generate ECP awareness during ANC and FWA visits

**Fig. 3:**
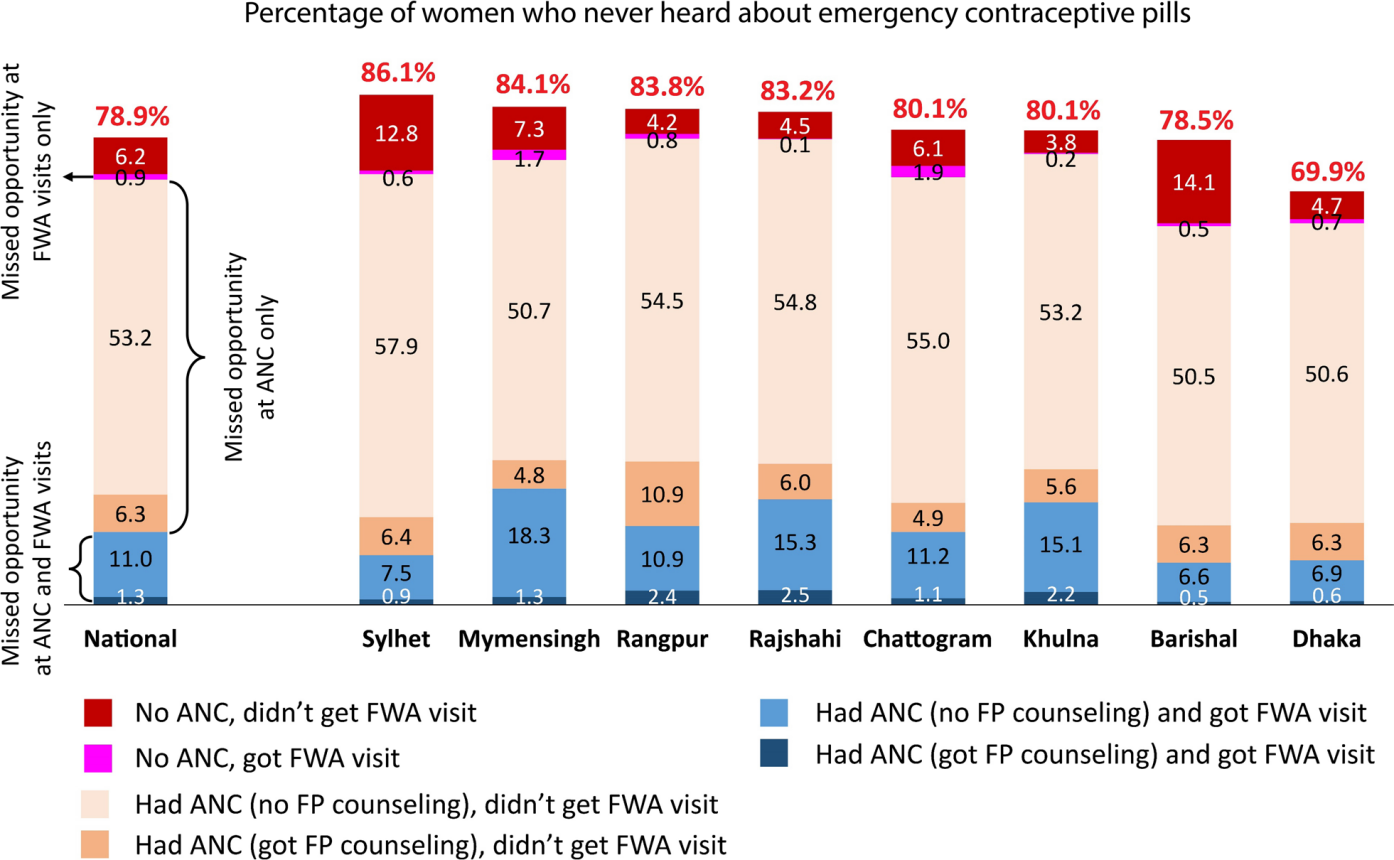
Percentage of women who never heard about emergency contraceptive pills among women aged 15–49 who gave live birth 3 years preceding the BDHS 2017–18

Nationally, 78.9% of women who gave live birth in the last 3 years preceding the survey were unaware of ECP (Fig. 3). However, only 6.2% of women were unexposed to ANC or FWA visits and remained unaware of ECP. The estimated missed opportunities in generating ECP awareness were 59.5% in ANC, 0.9% in FWA visits, and 12.3% in both ANC and FWA visits. The level of missed opportunity collectively in ANC and FWA visits was around 75% in all the divisions except for Dhaka (65.2%) and Barishal (64.4%).

### Prevalence and factors associated with ECP awareness

Only one-fifth (21.1%) of the women who had a live birth 3 years preceding the survey ever heard about ECP (Table 3). The prevalence of ECP awareness did not differ by FWA visit. Importantly, only one-third of the women who received FP counseling during ANC were aware of ECP. However, this was higher compared to women who did not receive FP counseling during ANC (34.2% vs. 20.2%). Urban women were more aware of ECP than rural women (31.9% vs. 17.1%). ECP awareness was monotonically increased by women’s and their husbands’ education and household wealth.

**Table 3:** Prevalence and factors associated with ECP awareness among women aged 15–49 who gave live birth three years preceding the BDHS 2017–18 (n = 5012)

Factors	Prevalence^1^	AOR	95% CI
Overall	21.1		
**Got FWA visit**			
No	21.5	Reference	
Yes	19.0	1.14	(0.89, 1.44)
**FP Counseling during last birth**			
Didn’t receive FP Counseling during ANC	20.2	Reference	
Received FP Counseling during ANC	34.2	1.53^**^	(1.19, 1.97)
Had no ANC during last birth	11.0	1.20	(0.77, 1.87)
**Type of residence**			
Urban	31.9	Reference	
Rural	17.1	0.77^*^	(0.61, 0.95)
**Administrative division**			
Dhaka	30.1	Reference	
Barishal	21.5	1.07	(0.75, 1.52)
Chattogram	19.9	0.59^**^	(0.42, 0.82)
Khulna	19.9	0.68^*^	(0.47, 0.99)
Mymensingh	15.9	0.68^*^	(0.48, 0.96)
Rajshahi	16.8	0.61^*^	(0.41, 0.90)
Rangpur	16.2	0.64^*^	(0.43, 0.94)
Sylhet	13.9	0.66^*^	(0.47, 0.91)
**Women’s current age**			
15–19	15.1	Reference	
20–24	22.4	1.31^*^	(1.01, 1.69)
25–29	23.0	1.45^*^	(1.08, 1.93)
30–34	22.6	1.50^**^	(1.11, 2.03)
35–49	18.8	1.49	(0.97, 2.28)
**Women’s education**			
No education or primary	9.7	Reference	
Secondary incomplete	18.2	1.48^**^	(1.17, 1.88)
Secondary complete or higher	43.9	3.18^**^	(2.35, 4.30)
**Watching television**			
Not at all	10.9	Reference	
Less than once a week	19.2	1.68^**^	(1.23, 2.29)
At least once a week	28.2	1.62^**^	(1.26, 2.09)
**Husband’s education**			
No education or primary	10.4	Reference	
Secondary incomplete	21.5	1.62^**^	(1.28, 2.05)
Secondary complete or higher	42.2	2.44^**^	(1.83, 3.24)
Unknown	10.6	0.88	(0.37, 2.05)
**Household wealth quintile**			
First	7.3	Reference	
Second	12.6	1.38	(0.94, 2.00)
Third	17.9	1.51^*^	(1.04, 2.18)
Fourth	24.3	1.56^*^	(1.05, 2.33)
Fifth	44.2	2.52^**^	(1.63, 3.89)

^**1**^Percentage of women heard about ECP among women aged 15–49 who gave live birth 3 years preceding the BDHS 2017–18; ^*^p < 0.05, ^**^p < 0.01; AOR: Adjusted odds ratio; CI: Confidence interval

The results of the multivariable regression suggest no significant association between FWA visit and ECP awareness (Table 3). However, a strong positive association between FP counseling during ANC and ECP awareness was noticed. The odds of ECP awareness was 53% higher (AOR: 1.53, 95% CI 1.19–1.97) among women who received FP counseling during ANC than those who did not receive FP counseling during ANC. The odds of ECP awareness was 3 times (AOR: 3.18, 95% CI 2.35–4.30) among women with secondary or higher levels of education in reference to women with no or primary level education. In addition, residing in urban areas, frequently watching television, higher educational level of husbands, belonging to middle or upper quintiles were associated with higher odds of ECP awareness.

### Missed opportunities for family planning counseling during ANC

**Fig. 4:**
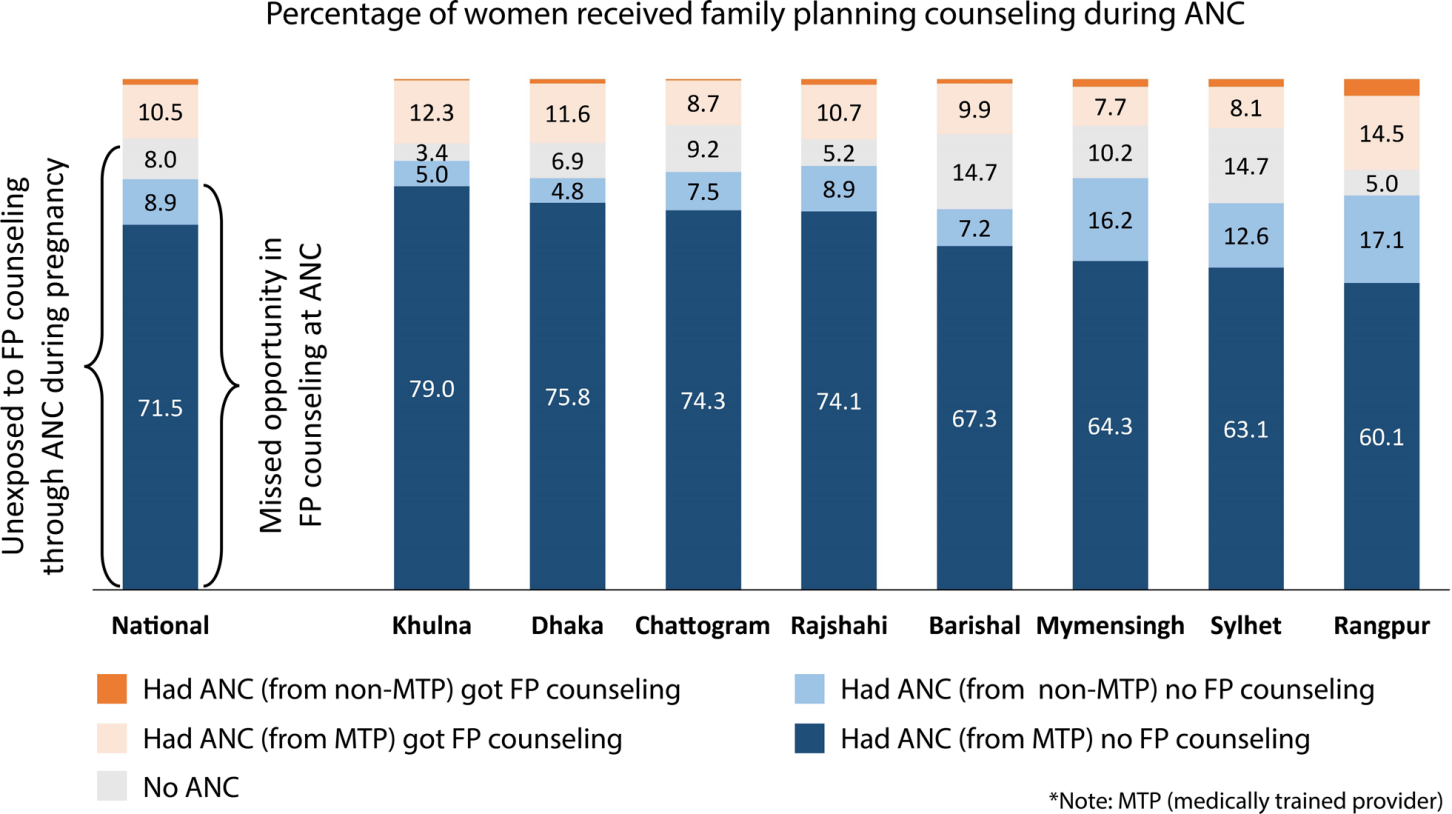
Percentage of women received family planning counseling during ANC for their most recent live birth among women aged 15–49 who gave live birth 3 years preceding the BDHS 2017–18 (n = 5012)

Figure 4 displays the status of FP counseling during ANC. The majority of women (88.4%) who gave their last live birth in 3 years preceding the survey remained unexposed to FP counseling through ANC during their last pregnancy. The missed opportunity in FP counseling during ANC was 80.4%. Devastatingly, missed opportunity from medically trained providers was 72%. Missed opportunity remains around 80% across divisions.

For readers’ convenience, we have addressed ANC-taker women as “women” while interpreting Table 4**.** The overall prevalence of missed opportunities in FP counseling during ANC (among ANC-takers) was 87.4% and it was above 80% across all the socio-demographic groups. The prevalence of missed opportunities was similar among women who received ANC from MTO and non-MTO providers. A higher prevalence of missed opportunities was observed among women with low education, first parity, residing in rural, and from bottom wealth quintile.

**Table 4:** Prevalence and factors associated with not receiving family planning counseling during ANC among women aged 15–49 who had ANC visits for the most recent live birth 3 years preceding the BDHS 2017–18 (n = 4604)

Factors	Prevalence^1^	AOR	95% CI
**Type of ANC provider**			
Medically trained provider	87.3	Reference	
Non-medically trained provider	88.8	0.93	(0.64, 1.37)
**Women’s education**			
Secondary complete or higher	81.0	Reference	
Secondary incomplete	88.9	1.91^**^	(1.46, 2.51)
No education or primary	90.3	2.23^**^	(1.61, 3.09)
**Parity**			
Third or higher	87.2	Reference	
Second	86.9	1.23	(0.93, 1.62)
First	88.1	1.53^**^	(1.15, 2.03)
**Type of residence**			
Urban	84.8	Reference	
Rural	88.5	1.20	(0.94, 1.52)
**Administrative division**			
Dhaka	86.6	Reference	
Chattogram	90.3	1.45	(1.00, 2.11)
Barishal	87.3	0.90	(0.58, 1.39)
Khulna	87.2	0.87	(0.59, 1.30)
Mymensingh	89.8	1.10	(0.72, 1.69)
Rajshahi	87.7	0.88	(0.55, 1.41)
Rangpur	81.2	0.50^**^	(0.34, 0.75)
Sylhet	89.0	1.02	(0.63, 1.64)
**Household wealth quintile**			
Fifth	82.4	Reference	
Fourth	87.3	1.29	(0.93, 1.78)
Third	88.4	1.38	(0.98, 1.93)
Second	89.7	1.49^*^	(1.01, 2.20)
First	89.8	1.56^*^	(1.03, 2.36)

^**1**^Percentage of women did not receive family planning counseling during ANC among women aged 15–49 who had ANC visits for most recent live birth 3 years preceding the BDHS 2017–18; ^*^p < 0.05, ^**^p < 0.01; AOR: Adjusted odds ratio; CI: Confidence interval

Results from the multivariable regression in Table 4 reveal the group of women whom we are significantly deprived of FP counseling during ANC. Unpleasantly, the odds of missed opportunities was not significantly lower among women who took ANC from MTO than non-MTO. The odds of missed opportunities was 2 times (AOR: 2.23, CI 1.61–3.09) among the women with no or primary education compared to women with secondary completed or higher education. Women of first parity had 53% greater odds of not receiving FP counseling than women with third or higher-order parities. The odds of missed opportunities were respectively 49% (AOR: 1.49, CI 1.01–2.20) and 56% (AOR: 1.56, CI 1.03–2.36) higher among women from the first and second wealth quintiles compared to women from the highest quintile.

## Discussion

### Main findings

Effective use of ECP could lessen the heavy burden of unintended conception in Bangladesh. Despite the high coverage of ANC and FWA visits, ECP awareness remains critically low here. The first objective of this study was to investigate the missed opportunities in generating ECP awareness through ANC and FWA visit programs. Using the national level data, we found huge missed opportunities in generating ECP awareness through ANC and FWA programs. In addition, regression analysis revealed that family planning counseling during ANC helped increase ECP awareness but the FWA program did not. The second objective was to assess the missed opportunities and sociodemographic discrimination in receiving FP counseling during ANC. We found a tremendously high level of missed opportunities in FP counseling during ANC; particularly among women with low education, lower parity, and poor socioeconomic status. These findings may aid in the development of a targeted service delivery model that could potentially improve the quality of FP counseling resulting in adequate knowledge and effective use of ECP among reproductive-aged women.

### Social inequity in ECP awareness

The deep-rooted sociocultural beliefs and norms often influence people’s perceptions of contraception, particularly in rural areas of LMICs where education is not optimal. Additionally, socio-religious values can limit discussions on contraception, further limiting awareness. We found substantial social inequity in ECP awareness. Women from rural areas, with low education, limited media exposure, and from lower wealth quintiles were less aware of ECP which is consistent with the earlier studies from BDHS 2014 [19, 20].

Despite being a patriarchal Muslim-majority LMIC, Bangladesh remarkably reduces its TFR by uplifting modern contraceptive use. The knowledge of modern contraceptive methods is now almost universal among Bangladeshi married women. Women who are not using a method, none are left unaware of contraceptive methods [27]. There is no differential in modern method use between rural and urban areas (54% vs. 55%) [27]. Thus, the urban–rural inequity in ECP awareness may be attributable more to the differentials in programmatic factors rather than the differentials in socio-cultural beliefs and norms between rural and urban. For example: less internet access and scarcity of social marketing campaigns could be the possible drivers of low ECP awareness among rural women [28]. Girls from rural areas and poor households are still vulnerable to child marriage, which limits their educational attainment. This disadvantageous group has high child wantedness. Thus, may have limited search for contraceptive methods which possibly left them unaware of ECP [29]. In addition, ECP is a costly FP method compared to other methods, making it less familiar to poor people.

### Missed opportunity to generate ECP awareness

The limited ECP awareness despite receiving ANC and FWA visits signals the fragile status of ECP counseling during these programs. FWA programs started in 1976 with 23,500 FWAs when the population was 78.8 million [18]. By 2017, the number of FWAs reduced to approximately 19,600 with the population increasing more than 2 times [17]. Thus, FWAs are now in serious human resource scarcity which may have severely affected the quality of counseling [30]. In a recent study providers mentioned human resource scarcity as one of the main reasons for limited FP counseling during ANC [31]. This human resource scarcity could be attributed to limited counseling on ECP despite high coverage of services uptake.

When the scope of counseling is limited, providers may choose a limited number of methods he/she is more convinced to discuss with women. Literature shows that provider bias often stems from their perception of contraception methods on future fertility [32, 33]. A systematic review from low and middle-income countries revealed widespread misconceptions among providers, including the abortifacient perception of ECP or that access to it would increase sexual activity among adolescents [32]. However, we cannot certainly conclude whether such perceptions from Bangladeshi providers restrict them from providing counseling on ECP in ANC and FWA visits.

### Missed opportunities for FP counseling in ANC

In addition to ECP, overall FP counseling is low during ANC. We found that even MTPs are not providing FP counseling, which warrants scrutinizing the ANC programs. Literature shows that providers referred to human resource scarcity, patient load within a short time, and women’s unwillingness to hear FP during ANC as the main reasons for not counseling [31]. Studies also showed that ANC providers from the Government Upazila Health Complex (UHC) perceived treating pregnancy complications as their main responsibility, and FP counseling is the responsibility of the Family Planning Division [31]. In addition, both providers from UHC and mothers perceived that the pregnancy period is not suitable for discussing FP [31].

Moreover, inadequate training of the providers could also be attributed to limited FP counseling. Bangladesh Health Facility Survey 2022 revealed the fragile status FP training. In 40% of public facilities that offer modern FP methods, all the interviewed staff never received any training in some aspect of FP [34]. Above this, only 20% of these facilities had at least one interviewed staff who received FP training in past 2 years [34].

### Sociodemographic discrimination in receiving FP counseling at ANC

In Bangladesh, most of the short-spaced, mistimed, and unwanted pregnancies are concentrated among less educated, rural, and poor women [35]. Our findings also reveal their limited FP counseling reception. FP conversation during ANC should be initiated by the provider, but in many cases, it is often initiated by clients (women) in Bangladesh. Thus, limited FP urge and inquiry from the disadvantageous (less educated, poor) mothers might subside FP conversation during ANC. In addition, educated women may better recall the FP counseling during ANC than less educated women because they have more urge to FP than less educated women. One possible reason for low FP counseling reception among low-parity mothers is that ANC providers find FP counseling easier for higher-parity women because they (women) have a fear of another child [31].

### Strength and limitations

The main strengths of this study include the use of DHS data that ensures the quality of the questionnaire, representativeness of the sample at the national and divisional levels, high response rate (99%), and data completeness (100%). The use of survey weight and survey design characteristics of the BDHS 2017–18 reduces the bias from the estimates and yields robust standard error of the estimates. This study may also be affected by recall bias. Only a small proportion of mothers have to recall a maximum of 3 years back to report whether they received FP counseling during ANC. The magnitude of the recall period bias is possibly similar for all sociodemographic groups because the recall period depends on the time since the last birth which is unlikely to vary among sociodemographic groups.

### Policy implication of findings

Findings highlight critical gaps and opportunities within Bangladesh’s reproductive health programs. The findings of this study involve two strong policy implications.

***Enhanced integration of FP counseling in ANC services:*** Findings reveal the catastrophic missed opportunity to provide FP counseling and generate ECP awareness during ANC visits. This underscores the need to enhance training and resources for healthcare providers involved in ANC services. Operational plans of the Health Sector Program should prioritize comprehensive FP counseling as a core component of ANC disregarding the sociodemographic characteristics of pregnant women.

***Reform and strengthening of the FWA Program:*** Findings reveal the ineffectiveness of the FWA program to improve ECP awareness which warrants re-evaluating and reinforcing the program’s training modules, outreach strategies, and accountability mechanisms.

## Conclusions

This study highlights the substantial missed opportunity to generate ECP awareness through ANC and FWA programs. Increasing human resources and provider knowledge of ECP can significantly contribute to offsetting some of the provider bias and thus improving ECP awareness among women [33]. Further investigation is also recommended to better understand the reasons for missed opportunities. Moreover, findings reveal the disadvantaged women who know less about ECP and are informed less about FP during ANC which may help policies reach the unreached women.

## Data Availability

This study used secondary data from Bangladesh Demographic and Health Survey 2017–18. The dataset can be obtained from the DHS website (https://dhsprogram.com/data/available-datasets.cfm) and it is publicly available upon reasonable request.

## Abbreviations

TFR: Total fertility rate
ECP: Emergency contraceptive pill
FP: Family planning
ANC: Antenatal care
FWA: Family welfare assistant
BDHS: Bangladesh demographic and health survey
MTP: Medically trained providers
UHC: Upazila Health Complex
